# The Gut Microbiota Can Provide Viral Tolerance in the Honey Bee

**DOI:** 10.3390/microorganisms9040871

**Published:** 2021-04-17

**Authors:** Christopher Dosch, Anja Manigk, Tabea Streicher, Anja Tehel, Robert J. Paxton, Simon Tragust

**Affiliations:** Department General Zoology, Martin-Luther-University Halle-Wittenberg, Hoher Weg 8, 06120 Halle (Saale), Germany; christopher.dosch@student.uni-halle.de (C.D.); anja.manigk@zoologie.uni-halle.de (A.M.); tabea.streicher@zoologie.uni-halle.de (T.S.); anja.tehel@zoologie.uni-halle.de (A.T.); robert.paxton@zoologie.uni-halle.de (R.J.P.)

**Keywords:** emerging infectious disease, DWV-B, immune defense, defensive symbiosis, microbiome

## Abstract

Adult honey bees host a remarkably consistent gut microbial community that is thought to benefit host health and provide protection against parasites and pathogens. Currently, however, we lack experimental evidence for the causal role of the gut microbiota in protecting the Western honey bees (*Apis mellifera*) against their viral pathogens. Here we set out to fill this knowledge gap by investigating how the gut microbiota modulates the virulence of a major honey bee viral pathogen, deformed wing virus (DWV). We found that, upon oral virus exposure, honey bee survival was significantly increased in bees with an experimentally established normal gut microbiota compared to control bees with a perturbed (dysbiotic) gut microbiota. Interestingly, viral titers were similar in bees with normal gut microbiota and dysbiotic bees, pointing to higher viral tolerance in bees with normal gut microbiota. Taken together, our results provide evidence for a positive role of the gut microbiota for honey bee fitness upon viral infection. We hypothesize that environmental stressors altering honey bee gut microbiota composition, e.g., antibiotics in beekeeping or pesticides in modern agriculture, could interact synergistically with pathogens, leading to negative effects on honey bee health and the epidemiology and impact of their viruses.

## 1. Introduction

Gut-associated microbial communities are ubiquitous in animals, and their presence can affect important host traits [1,2]. Social bees are no exception, featuring a recurring set of bacterial species clusters (phylotypes) in their gut [3]. In the eusocial Western honey bee (*Apis mellifera*), the gut microbiota is dominated by up to nine bacterial phylotypes [4,5], which are acquired by adult honey bees after emergence through contact with nest material and nestmates [6,7]. Several studies (reviewed in [8,9,10]) have shown that the presence of the characteristic, normal honey bee gut microbiota, either in its entirety or of single members, is related to bee health and physiology and likely plays a role in protection against a range of parasites and pathogens.

Honey bees are important pollinators [11] that have suffered elevated mortality in recent years [12]. Overwintering honey bee colony losses have been linked to the presence of the exotic invasive ectoparasitic mite *Varroa destructor* and the viruses it transmits [13,14,15,16], particularly deformed wing virus (DWV) [15], which has become panzootic in honey bee populations where *V. destructor* has been introduced [16,17,18,19]. The two widespread genotypes of DWV, genotypes A and B, are both highly virulent pathogens of *A. mellifera* when transmitted by *V. destructor* or experimentally by injection [20], though DWV is also readily transmitted orally [17].

To date, only a single study surveying the presence of gut microbiota in larvae of the Asian honey bee *Apis cerana* reported a lower abundance of two representatives of the associated microbiota in sacbrood virus-infected colonies [21], suggesting that the gut microbiota may protect honey bees from viruses. However, we currently lack functional assays demonstrating the microbiota’s beneficial effects against viruses, either in larvae or in adults. We, therefore, set out to test whether the presence of the honey bee gut-associated microbial community in adult honey bees influences DWV-B titer and virulence in a full factorial laboratory experiment, manipulating both microbiota and virus.

## 2. Materials and Methods

### 2.1. Experimental Procedures

To obtain adult bees, brood frames from two colonies (Section 2.2) were brought into the laboratory and incubated in a chamber at 35 °C and 50% relative humidity overnight, from which bees eclosed naturally. The next day, day 0 of the experiment, newly eclosed bees that largely lack the honey bee characteristic gut microbiota [6,7] were transferred into autoclaved metal cages (18 individuals per cage, 16 cages per colony, the total number of cages: 32, the total number of bees: 576) with access to a sterile 1:1 sucrose-water solution in an incubator at 35 °C and 50% relative humidity.

On day 1, half of the cages (*n* = 16 cages, 288 bees) were given access to a gut homogenate for 24 h (group: microbiota+) to establish the characteristic bee gut microbiota (see Section 2.3 for gut homogenate preparation). This method of establishing the gut microbiota was chosen as it yields bees with robust gut communities similar to those of normal bees sampled from hives [7,22,23]. The other half of the cages (*n* = 16 cages) retained the sterile sucrose-solution as their food source (group: microbiota-), which limits the establishment of the normal gut microbiota [24].

On day 6 of the experiment, three experimental bees were randomly removed from each cage (except cage B1 in the group microbiota- from which only two bees were removed, as three bees had already died, Table S1) and stored in 1.5 mL tubes containing 95% ethanol at −20 °C [7] for later bacterial abundance estimation (Section 2.4 and Section 2.5) in a subset of these sampled bees (*n* = 1 bee per cage, total bees: 32). As a reference to experimental microbiota+ and microbiota− bees, eight hive bees taken from brood frames from each of the two source colonies were collected and similarly stored for later quantification of bacterial abundance in a subset of these hive bees (*n* = 4 for colony G1 and *n* = 2-3 for colony 5.1). Bacterial abundance was determined of either specific members of the bee gut microbiota (*Gilliamella apicola*, *Frischella perrara*, *Snodgrasella alvi*, *Bartonella apis*, *Bifidobacterium asteroids*, *Lactobacillus Firm-4*, *Lactobacillus Firm-5*) or universally all bacteria (16S) via quantitative polymerase chain reaction (qPCR). The remaining experimental bees in the cages at day 6 were starved for 3–4h before individually feeding all bees in half of the microbiota+ and the microbiota- cages (*n* = 8 cages each) with 5 µL of a DWV-B inoculum (concentration 2 × 10^8^ genome equivalents per µL; see Section 2.6 for preparation of the inoculum) (groups: microbiota+|virus+ and microbiota−|virus+), while all bees in the other half of the cages were individually fed with sterile 5 µL 1:1 sucrose-water solution (groups: microbiota+|virus− and microbiota−|virus−, *n* = 8 cages each) (see Section 2.7 for details on individual feeding procedures).

On day 11 of the experiment, a time point after successful establishment of DWV-B infection but likely before virus-induced mortality [20,25], we again collected randomly two to three bees from each cage and stored them individually in 1.5 mL tubes at −80 °C for later quantification of the viral titer (Section 2.8) in a subset of these sampled bees (*n* = 1 bee per cage except for one cage in the group microbiota−|virus+ in which no bee was sampled as almost all had died, total *n* = 31). Thereafter, bee survival was checked daily for a total of 31 experimental days, with dead bees being removed daily from the cages. During this time, sterile 1:1 sucrose-water solution was the only food source given to the bees.

### 2.2. Honey Bee Origin

Honey bees originated from two colonies (5.1 and G1) in the General Zoology apiary at Martin Luther University Halle-Wittenberg, Germany. The colonies were treated with Bayvarol^®^ and were then, two weeks after treatment with Bayvarol^®^ and three weeks before the start of the experiments, screened for the presence of six common bee viral targets: deformed wing virus A and B (DWV-A and DWV-B), black queen cell virus (BQCV), chronic bee paralysis virus (CBPV), sacbrood virus (SBV), and slow bee paralysis virus (SBPV) using the methodology described below (Section 2.8). None of the viral targets could be detected in the colonies up to a quantitative polymerase chain reaction (qPCR) cycle (Ct) of 40 at the time of sampling.

### 2.3. Preparation of the Gut Homogenate for Microbiota Feeding

The gut homogenate used to inoculate experimental bees with the characteristic gut microbiota via feeding was freshly prepared from hive workers on the day of inoculation (day 1 of the experiment). To prepare the gut homogenate, ten bees were collected from brood frames of each of the two source colonies, brought into the laboratory, cold anesthetized on ice, and then sacrificed by dissecting their gastrointestinal tracts (gut sections: midgut/ventriculus, ileum, and rectum, excluding the crop) under sterile conditions near a flame. The dissected gastrointestinal tracts were then pooled per source colony, homogenized in 3 mL sterile 1:1 sucrose-water with a sterile pestle, and finally diluted 3:8 with sterile 1:1 sucrose-water solution to obtain the gut homogenate. This mixture was then given to experimental bees in the cages via bulk feeding, i.e., providing them with a feeding tube containing the mixture for 24 h.

### 2.4. Microbiota Abundance Estimation

For bacterial abundance estimation in experimental bees collected on day six of the experiment and in-hive bees removed directly from brood frames of the source colonies, we followed the methods outlined in [7] for dissection and DNA extraction (Section 2.4.1) of individual bee guts and then performed quantitative polymerase chain reaction (qPCR) assays (Section 2.4.2 and Section 2.4.3) with primers (Table S2) targeting the 16S rRNA gene of either specific members of the bee gut microbiota (*Gilliamella apicola*, *Frischella perrara*, *Snodgrasella alvi*, *Bartonella apis*, *Bifidobacterium asteroids*, *Lactobacillus Firm-4*, *Lactobacillus Firm-5*) or universally all bacteria (16S).

#### 2.4.1. Dissection of Honey Bee Guts and DNA Extraction

For dissection, individual bees (experimental microbiota− and microbiota+ bees and hive bees from the source colonies) stored in 95% ethanol at −20 °C were first dried on a clean laboratory tissue for 4–8 min, and then the gut region, including the midgut/ventriculus, the ileum, and the rectum was dissected out of each bee under sterile conditions near a flame. For DNA extraction using a cetyltrimethylammonium bromide (CTAB) bead-beating method, dissected guts were individually placed in 2 mL tubes together with 730 µL 2% CTAB-buffer (100 mL of 1 M Tris HCl adjusted to pH 8, 20 mL of 0.5 M EDTA, 81.8 g NaCl, 20 g CTAB, and ddH_2_0 to 1 L), 250–350 µL 0.1 mm silica zirconia beads (BioSpec), and 20 µL Proteinase K (Sigma). Samples were then homogenized with a Tissue Lyser LT (Qiagen) at full speed for 2 min, placed on ice for 1 min, and bead-beaten again for 2 min before incubating the samples overnight at 56 °C. Thereafter, 750 µL phenol-chloroform-isoamyl alcohol (25:24:1) was added, tubes were mixed by inverting and placed on ice for at least 2 min before centrifugation at 7000 rpm for 15 min at 4 °C. Then, the DNA in the aqueous phase was alcohol precipitated (twice with 200 µL 2-propanol), washed (200 µL ice-cold 70% Ethanol), and air-dried prior to resuspension in 50 µL nuclease-free water. The concentration of extracted DNA was then determined with an Epoch™Microplate Spectrophotometer (BioTek), and the DNA was stored at −80 °C.

#### 2.4.2. qPCR for Microbial Abundance Quantification

For the quantification of either specific members of the bee gut microbiota or universally all bacteria, duplicate qPCRs were performed, and the mean Ct value was used. qPCRs were performed in a Bio-Rad C1000 thermal cycler (Bio-Rad, Munich, Germany) for the specific gut microbiota members in 10 µL reactions with 5 µL SYBRgreen Sensimix (Bioline, Luckenwalde, Germany), 3.6 µL ddH2O, 0.2 µL of each primer (10 µM) and 1 µL 1:10 diluted template DNA with the following program: 10 min at 95 °C, followed by 40 cycles of 15 s at 95 °C, 30 s at 60 °C and 30 s at 72 °C. The qPCRs targeting universally all bacteria were run in a QuantStudio 3 Real-Time PCR System (ThermoFisher, Waltham, MA, USA) in 20 µL reactions with 10 µL Fast SYBR-Green Master Mix (ThermoFisher, Waltham, MA, USA), 5 µL ddH2O, 1.25 µL of each primer (10 µM) and 2.5 µL template DNA (65 ng/µL) with the following program: 95 °C for 20 s followed by 40 cycles at 95 °C for 3 s and 60 °C for 30 s.

All qPCRs included a duplicate dilution series of an external DNA standard (10^9^-10^3^ 16S rRNA gene copies), which was used to generate calibration curves for the quantification of the bacterial target. External standards for specific members of the bee gut microbiota, except for *Snodgrasella alvi*, consisted of purified qPCR fragments that were generated in qPCRs with primers listed in Table S2 as outlined above using DNA acquired from the German Collection of Microorganisms and Cell Cultures GmbH, DSMZ, Braunschweig (*Gilliamella apicola*: DSM 104097; *Frischella perrara*: DSM 104328; *Bartonella apis*: DSM 29779; *Bifidobacterium asteroids:* DSM 20089; *Lactobacillus Firm-4*: DSM 26255 *Lactobacillus Firm-5:* DSM 26256). To create external DNA standards for *Snodgrasella alvi* and universally all bacteria, we cultured *Snodgrasella alvi* (strain DSM 104735, DSMZ, Braunschweig) and *Escherichia coli* (strain YM109, obtained from PD Dr. Silvio Erler, Leibnitz Institute Braunschweig), extracted their DNA as described in Section 2.4.1. and then used as external standards the purified PCR fragments generated in a 20 µL PCR reaction consisting of 8 µL 5× Buffer (Promega), 9.2 µL ddH2O, 0.6 µL dNTP, 0.5 µL of each primer (10 µM), 0.2 µL Taq (Promega GoTaq) and 1 µL template (1:10 diluted DNA) with the following program: 95 °C for 2 min, 40 cycles at 95 °C for 30 s, 60 °C (*Snodgrasella alvi*)/56 °C (*Escherichia coli*) for 45 s and 72 °C for 30 s with a final 72 °C for 5 min. To calculate the starting concentration of the external standard (copy number/µL), the equation in [26] was used, and the mass concentration of qPCR/PCR fragments was measured with an Epoch™Microplate Spectrophotometer (BioTek, Winooski, VT, USA) directly after purification.

#### 2.4.3. Quality Control of qPCRs for Bacterial Abundance Estimation

The following quality control checks were run for each qPCR 96-well reaction plate. To check that the correct template had been amplified, qPCR products were denatured for one minute at 95 °C, cooled to 55 °C for one minute, and then a melting profile was generated from 55 to 95 °C (0.5 °C per second increment). In addition, two duplicated no template controls (negative control) were included on 96-well plates detecting universally all bacteria or *Snodgrasella alvi*, while one duplicated no template control was included in the other 96-well plates.

### 2.5. Bacterial Cultivation

To create external DNA standards for bacterial abundance estimation of *Snodgrasella alvi* and universally all bacteria (Section 2.4.2), *Snodgrasella alvi* (dry culture) and *Escherichia coli* (single colony-forming units on an agar plate obtained from PD Dr. Silvio Erler, Leibnitz Institute Braunschweig) were grown in liquid culture for 24 h at 30 °C in 5 mL Caso medium (Roth) and LB-medium (10 g Tryptone, 5 g yeast extract in 1L MilliQ-water), respectively. The culture of *E. coli* was performed under a normal atmosphere while the culture of *Snodgrasella alvi* was performed under an 8–10% CO_2_ atmosphere, placing the liquid culture in an airtight 2 L plastic container (Emsa clip and close) and adding an Anearocoult^®^ C sachet (Merck) according to the manufacturer’s instructions.

### 2.6. Virus Propagation and Preparation of Virus Inoculum

To prepare the experimental DWV-B virus inoculum and to propagate DWV-B, we injected 1 µL of the DWV-B inoculum from [27] into the hemolymph of white-eyed pupae from colony G1 at a concentration of 1 × 10^5^ DWV-B virus particles in 1 µL and incubated them for three days in an acrylic box at 35 °C and 50% relative humidity. After three days, three pupae were pooled at a time, crushed in 900 µL of 0.5 M potassium phosphate buffer (pH 8.0) using a sterile plastic pestle, mixed thoroughly, and centrifuged for 2 min at 15,000× *g*. The supernatant was kept as the new inoculum and stored at −80 °C while precipitated honey bee pupal debris was discarded. This newly created inoculum was then checked for the presence of six common bee viral targets: deformed wing virus A and B (DWV-A and DWV-B), black queen cell virus (BQCV), chronic bee paralysis virus (CBPV), sacbrood virus (SBV), and slow bee paralysis virus (SBPV) and the concentration of DWV-B quantified using the methodology described below (Section 2.8). Except for DWV-B, none of the other viral targets could be detected up to a qPCR cycle (Ct) of 40 in this newly created DWV-B inoculum. The inoculum was not checked for the presence of honey bee gut microbiota members and for other honey bee pathogens. Thus, we cannot completely rule out the possibility that feeding of the virus inoculum to bees in the group microbiota−|virus+ at day six of the experiment might have led to the inadvertent establishment of the honey bee gut microbiota, and we cannot completely rule out that mortality in virus-exposed bees can solely be attributable to the action of virus exposure alone.

### 2.7. Virus Feeding

To ensure full uptake of the virus inoculum, all bees were fed individually on day six. For this, bees were removed singly from each cage using forceps, their wings were grasped between the thumb and index finger to expose the mouthparts, and then 5 µL of the virus inoculum (concentration 2 × 10^8^ genome equivalents per µL) was administered to bees in the experimental group microbiota+|virus+ and microbiota−|virus+ group using a pipette [28], before putting bees into new sterile cages according to their treatment. Bees in experimental groups microbiota+|virus− and microbiota−|virus− were similarly fed but exchanging the virus inoculum with a 1:1 sterile sucrose-water solution.

### 2.8. Virus Detection and Quantification

For viral detection in honey bee source colonies (Section 2.2) as well as the experimental DWV-B inoculum (Section 2.6) and for DWV-B quantification in the experimental DWV-B inoculum (Section 2.6) as well as in bees collected on day 11 of the experiment, we followed the methods outlined in [27] using RNA extraction, cDNA synthesis, and qPCR with primers listed in Table S2.

#### 2.8.1. RNA Extraction and cDNA Synthesis

To test whether the honey bee source colonies were free of virus infection, we collected at random 15 pupae from a brood frame per colony, pooled and snap-froze them on dry ice, then crushed them in a plastic RNAse-free mesh bag (BioReba, Reinach, Switzerland) with 7.5 mL of diethylpyrocarbonate (DEPC)-treated water, and recovered 100 µL of the homogenate from beyond the BioReba mesh for RNA extraction. To screen the experimental DWV-B virus inoculum for the presence of other honey bee viruses and for quantification of DWV-B titers in the inoculum, we used 100 µL of the inoculum for RNA isolation. For RNA extraction of bees arising from the experiment, whole bees stored at −80 °C were crushed in 500 µL RLT-Buffer using a plastic pestle, and 100 µL of the homogenate was used for RNA extraction.

RNA was extracted using an RNeasy mini kit (Qiagen, Hilden, Germany) following the manufacturer’s instructions in a QIAcube robot (Qiagen). cDNA was synthesized from RNA extracts using oligo(dT)_18_ primers (Thermo Scientific, Waltham, MA, USA) and reverse transcriptase (M-MLV and Revertase, Promega, Mannheim, Germany) following the manufacturer’s instructions. For cDNA synthesis, 800 ng of RNA was used, and the resultant cDNA was diluted 1:10 prior to use in qPCRs.

#### 2.8.2. qPCR for Viral Detection

For viral detection in the two honey bee source colonies (Section 2.2) and in the experimental DWV-B virus inoculum (Section 2.6), duplicate qPCRs per sample were performed in a Bio-Rad C1000 thermal cycler (Bio-Rad, Munich, Germany), using SYBRgreen Sensimix (Bioline, Luckenwalde, Germany) and the primers in Table S2 with the following program: five minutes at 95 °C, followed by 40 cycles of 10 s at 95 °C, 30 s at 57 °C and 30 s at 72 °C.

#### 2.8.3. qPCR for Viral Quantification

For absolute viral quantification of DWV-B in the experimental DWV-B virus inoculum (Section 2.6) and the bees arising from the experiment, duplicate qPCRs were performed with primers in Table S2, and the mean Ct value was used. The qPCR included a duplicate dilution series of an external DNA standard (10^10^–10^3^ viral particles), which was used to generate calibration curves for the quantification of the virus target. The external DNA standard consisted of purified PCR fragments which were generated using a 20 µL PCR reaction with 8 µL 5X Buffer (Promega), 9.2 µL ddH2O, 0.6 µL dNTP, 0.5 µL of each primer (10 µM), 0.2 µL Taq (Promega GoTaq, Mannheim, Germany) and 1 µL template 1:10 diluted cDNA of the DWV-B inoculum with the following program: 95 °C for 2 min, 40 cycles of at 95 °C for 30 s, 57 °C for 45 s and 72 °C for 30 s, with a final 72 °C for 5 min. To calculate the starting concentration of the external standard (copy number/µL), the equation in [26] was used, and the mass concentration of qPCR/PCR fragments was measured with an Epoch™Microplate Spectrophotometer (BioTek, Winooski, VT, USA) directly after purification.

#### 2.8.4. Quality Control of qPCRs for Viral Detection and Quantification

The following quality control checks were run for each qPCR 96-well reaction plate. To check that the correct template had been amplified, qPCR products were denatured for one minute at 95 °C, cooled to 55 °C for one minute, and then a melting profile was generated from 55 to 95 °C (0.5 °C per second increment). To check for RNA degradation, error in RNA extraction, or failure in cDNA synthesis, *A. mellifera* β-actin was also amplified for all of the samples as a honey bee internal reference marker using primers in Table S2 and the same qPCR program as for viral detection. In addition, a no template control (negative control) and a virus-infected (positive control) sample were included on each 96-well plate for virus detection, while the positive control was omitted for viral quantification.

### 2.9. Statistical Analyses

All statistical analyses were performed with the R statistical programming language (Version 3.6.1, [29]). Bacterial abundance or viral titer of samples that did not yield an amplification in the estimation via qPCR up to cycle 40 were set to zero. Survival data were analyzed with a Cox mixed-effects model (COXME, package “coxme”, [30]), with animal treatment (four levels: microbiota+|virus+, microbiota+|virus−, microbiota−|virus+, microbiota−|virus−) as fixed predictor and experimental cage as well as source colony as significant random factors (random effect cage: LR-test, χ^2^ = 15.126, df = 1, *p* < 0.001; random effect colony: LR-test, χ^2^ = 4.013, df = 1, *p* < 0.045). Bacterial abundance (log_10_ transformed and a value of one added to all values for the transformation) of specific members of the bee gut microbiota and universally all bacteria were analyzed in separate linear mixed-effects models (LMM, package “lme4”, [31]) with source colony as a random factor and bee origin/treatment groups (three levels: hive bees from the source colonies, experimental microbiota+, and microbiota− bees) as a fixed factor. Viral titers in bees exposed to the virus and not exposed to the virus were similarly analyzed in two different models. These models included colony source as a random factor and animal treatment (two levels: microbiota+|virus+ and microbiota−|virus+ for the model with virus-exposed bees and two levels: microbiota+|virus− and microbiota−|virus− for the model with bees not exposed to the virus) as fixed factors. To assess the significance of predictors, models were compared to null (intercept only) models using likelihood ratio (LR) tests. Pairwise comparisons between factor levels of a significant predictor were performed using pairwise post-hoc tests, adjusting the family-wise error rate according to the method of Westfall (package “multcomp” [32]).

Model assumptions of LMMs were checked with diagnostic tests and plots implemented in the package “DHARMa” [33]. No model diagnostic test or plot is currently available for Cox mixed-effects models (personal communication with package developer COXME: Terry Therneau). Statistical output tables for bacterial abundance and viral titer were created with the package “sjPlot” [34]. Survival curves for bee treatment (groups: microbiota+|virus−, microbiota+|virus+, microbiota−|virus− and microbiota−|virus+) based on Kaplan–Meier estimates were plotted with package “ggplot2” [35] and package “ggkm” [36], while bacterial abundance and viral titer were plotted with package “ggplot2” only. Description of the numbers of bees at risk (alive), the cumulative number of dead bees, and the cumulative number of censored bees at crucial time points during the experiment (day 0, 6, 7, 11, 12, 30, and 31) were generated with the package “survminer” [37].

## 3. Results

**Figure 1:**
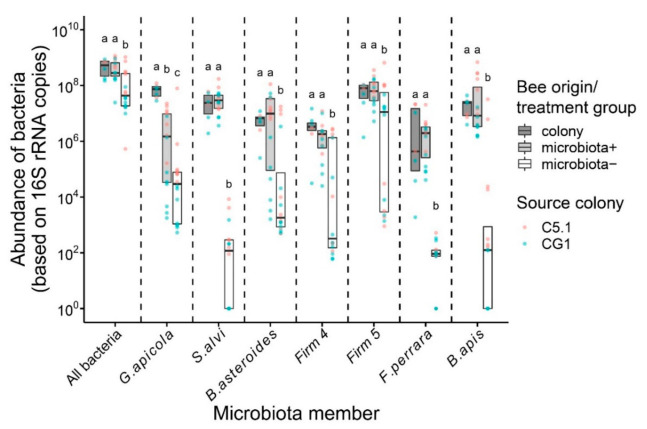
Abundance of six honey bee gut microbiota members (*G. apicola*, *F. perrara*, *S. alvi*, *B. apis*, *B. asteroids*, *Lactobacillus group Firm-4,* and *Firm-5*) and universally all bacteria (16S) in experimental bees at day six. Experimental bees were either fed bee gut microbiota (microbiota+) or not (microbiota−) at adult emergence to establish the normal gut microbiota. Bees directly out of source colonies (colony) served as a comparison. Lower case letters indicate statistically significant differences at α = 0.05 within groups separated by dashed lines. Boxplots show the median and interquartile range. Points represent individual bees, and colors indicate bee source colony of origin.

Experimental establishment of the gut microbiota in freshly emerged (1 day of age) microbiota+ bees led to the establishment of normal gut microbiota. Experimental microbiota+ bees did not differ in total abundance of gut bacteria and specific honey bee gut microbiota members in comparison to hive bees from their source colonies, except for the microbiota member *Gilliamella apicola* (Figure 1; Table S3). Experimental prevention of gut microbiota establishment in microbiota− bees led to bees that had a significantly lower abundance of gut bacteria and specific honey bee gut microbiota members compared to both experimental microbiota+ bees and hive bees form source colonies (Figure 1; Table S3). This indicates the successful establishment of the normal gut microbiota in experimental microbiota+ bees, while microbiota− bees were dysbiotic.

**Figure 2:**
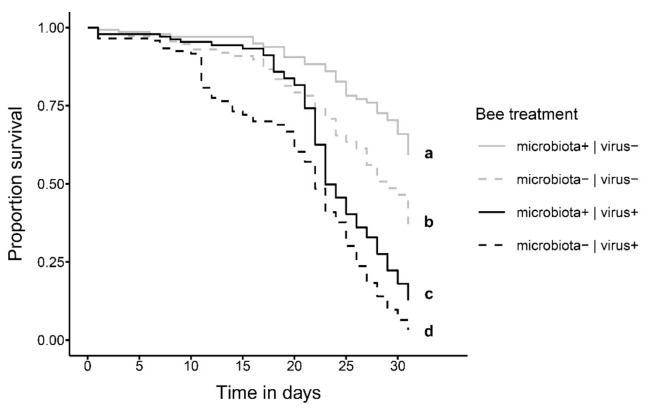
Survival of bees shown as Kaplan–Meier estimates over the course of the experiment (31 days) that were either fed a bee hindgut homogenate to establish the gut microbiota (microbiota+, solid lines) or not (microbiota−, dashed lines) at day 1 of adult emergence and were then either fed a DWV-B virus inoculum (virus+, black lines) or not (virus−, gray lines) at day 6 of the experiment. Small letters indicate statistically significant differences at α = 0.05 in a Cox mixed-effects model.

Bee survival was significantly affected by the presence of the normal gut microbiota and oral exposure to DWV-B at 6 days of age (Figure 2; Table S4; COXME, LR-test, χ^2^ = 31.672, df = 3, *p* < 0.001). Bees that were not exposed to the virus survived best, with microbiota+|virus− bees being only half as likely to die than microbiota−|virus− bees (hazard ratio: 0.52; post-hoc comparison: *p* < 0.001). Bees exposed to the virus survived significantly worse, with microbiota+|virus+ bees, again being only half as likely to die as microbiota−|virus+ bees (hazard ratio: 0.53; post-hoc comparison: *p* = 0.033). This indicates an overall negative effect of viral exposure on honey bee survival but a beneficial effect of the honey bee gut microbiota in reducing the viral impact on host honey bees.

**Figure 3:**
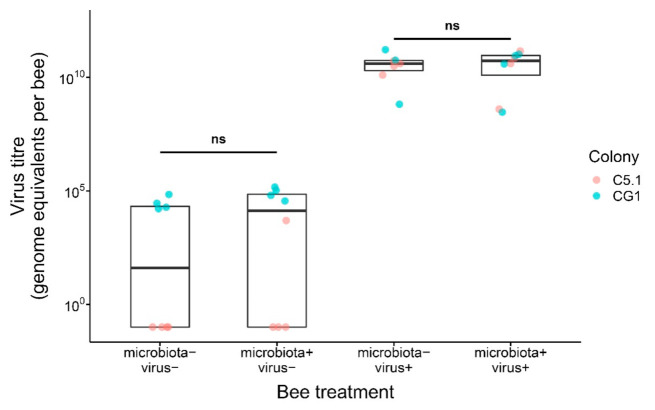
DWV-B virus titer at day 11 in experimental microbiota+ and microbiota- bees that were either fed a virus inoculum (virus+) or not (virus−) at day 6. Non-significant differences are indicated by “ns” (*p* > 0.05). Boxplots show the median and interquartile range. Points represent individual bees, and colors indicate bee source colony of origin.

Irrespective of whether or not the normal gut microbiota was established in bees, feeding of DWV-B virus to bees resulted in similar, high viral titers in microbiota+|virus+ and microbiota−|virus+ bees at day 11 of the experiment (Figure 3; Table S5; LMM, LR-test, χ^2^ = 0.136, df = 1, *p* = 0.712). Experimental bees that were not fed the virus showed only a low background infection, especially bees originating from source colony G1. Viral titer did not differ statistically in microbiota+|virus− and microbiota−|virus− bees (Figure 2, b; Table S4; LMM, LR-test, χ^2^ = 3.270, df = 1, *p* = 0.071).

## 4. Discussion

We found that experimental establishment of the characteristic gut microbiota was associated with improved honey bee survival when hosts were orally challenged in the laboratory with DWV-B virus. Our study, therefore, suggests that the beneficial effect of the honey bee gut microbiota against parasites and pathogens (reviewed in [8,9,10]) also holds for honey bee viruses.

The results of our study potentially even underestimate the beneficial effect of the honey bee gut microbiota upon virus exposure. Our microbiota− bees showed bacterial abundances in excess of 10^6^ cells for universally all gut bacteria, and for some of the specific honey bee gut microbiota members, a threshold often used to designate honey bees as microbiota-depleted [7,22,23,24]. Had we more successfully removed gut microbiota from our microbiota bees through, for example, removal of pupae from their frames before they had eclosed [7,24], we may have witnessed an even greater beneficial effect of microbiota on host bee survival. Future studies should evaluate the effect of viral exposure on microbiota-depleted bees.

We found that viral titers reached similarly high levels in virus-exposed bees irrespective of whether bees carried the characteristic gut microbiota or not. Higher fitness (survival) of bees with an established characteristic gut microbiota without control of parasite loads potentially suggests that the honey bee gut microbiota confers viral tolerance. Viral tolerance has been inferred in previous studies investigating varroa mite-resistant honey bees [38,39] (see also [40]), but an involvement of the gut microbiota has so far not been suggested. In contrast to resistance, i.e., the ability of a host to limit parasite load, tolerance is the ability of a host to limit the negative fitness effects of a given parasite load [41,42,43]. Host-associated microorganisms are known to modulate tolerance to infection in other animals [44,45], likely through processes that protect hosts directly or indirectly from pathogen damage or that maintain energy homeostasis [46]. The honey bee gut microbiota has marked effects on host physiology that might have contributed to damage repair or maintenance of energy homeostasis in our experimental setup, e.g., stimulation of the host immune system [47,48]. The honey bee gut microbiota also influences metabolic activities that contribute to honey bee nutrition and promote host weight gain [23,24]. Especially host diet deserves more attention since it has repeatedly been shown to affect disease tolerance in experimental studies on insects [41,49]. Future studies will have to determine the exact mechanistic basis of gut microbiota mediated viral tolerance in honey bees together with its generality for other honey bee viruses and its relevance in other bee species. Considering the presence of shared as well as host-specific bacterial phylotypes in bees [3], this might provide insights into the impact of the well-documented spill-over of viruses from honey bees to wild bees, especially bumblebees [50,51,52,53,54,55,56].

## 5. Conclusions

Many direct links between gut microbiota, disease, and dietary resources have been uncovered in animals in general and in the honey bee specifically ([57] and references therein). These links encompass factors that have been inferred in bee declines around the world [58], i.e., stressors from parasites, lack of dietary resources, and pesticides. The results of our study, together with the existence of potential indirect links [57], e.g., the influence of pesticides on the honey bee gut microbiota [59] and the influence of beekeeping practices [60,61] with likely percolating effects on nutrition and disease, highlight the need for integrated research investigating a multitude of factors in order to better understand the epidemiology and impact of honey bee viruses on their hosts. This is especially important since host tolerance, from a theoretical perspective, can increase parasite fitness and thus prevalence [62] and, from an applied perspective, could together with recently developed microbe based techniques [63] lead to better beekeeping practices, land use, and management that target maintenance of the honey bee gut microbiota to reduce honey bee colony losses.

## Data Availability

Data supporting the reported results and code to reproduce figures and analyses in R can be found as Supplementary material.

## Supplementary Materials

The following are available online at https://www.mdpi.com/article/10.3390/microorganisms9040871/s1, Table S1: Overview of the number of experimental bees at different time points of the experiment; Table S2: Primers and standard curve characteristics; Table S3: Output of statistical analyses for gut microbiota abundance; Table S4: Output of statistical analysis of experimental bee survival; Table S5: Output of statistical analyses of viral titer; Data analyses and script file: R code to reproduce figures and analyses; Data Figure 1: Raw data of gut microbiota abundance; Data Figure 2: Raw data of experimental bee survival; Data Figure 3: Raw data of viral titer.
