# Supplementary material for: The Gut Microbiota Can Provide Viral Tolerance in the Honey Bee
Source: Microorganisms. 2021 Apr 17;9(4):871. doi: 10.3390/microorganisms9040871 (PMC8072606; doi:10.3390/microorganisms9040871)
Supplement: Supplementary file 1 [file microorganisms-09-00871-s001.zip › microorganisms-1182355-supplementary/Supplementary material/Dosch et al. supplementary material Table S1.pdf]

Table S1. Overview of the number of experimental bees at risk (alive), the cumulative number of dead bees and the cumulative number of censored bees at day 0, 6, 7, 11, 12, 30, and 31 of the experiment according to source colony, cage and experimental treatment.

Colony	Cage	Treatment		Number of bees at risk (alive)							Cumlative number of dead bees							Cumlative number of censored bees						
			Day	0	6	7	11	12	30	31	0	6	7	11	12	30	31	0	6	7	11	12	30	31
C5.1	C1	microbiota+ | virus-		18	18	15	15	12	6	6	0	0	0	0	0	6	7	0	3	3	6	6	6	11
C5.1	C2	microbiota+ | virus-		18	17	14	14	11	8	8	0	1	1	1	1	4	4	0	3	3	6	6	6	14
C5.1	C3	microbiota+ | virus-		18	18	15	15	12	10	10	0	0	0	0	0	2	3	0	3	3	6	6	6	15
C5.1	C4	microbiota+ | virus-		18	18	14	14	11	10	10	0	1	1	1	1	2	2	0	3	3	6	6	6	16
CG1	H1	microbiota+ | virus-		18	18	15	15	12	9	7	0	0	0	0	0	3	6	0	3	3	6	6	8	12
CG1	H2	microbiota+ | virus-		18	18	15	15	12	10	9	0	0	0	0	0	3	4	0	3	3	6	6	6	14
CG1	H3	microbiota+ | virus-		18	17	11	10	8	4	3	0	1	1	2	2	7	7	0	6	6	8	8	8	11
CG1	H4	microbiota+ | virus-		18	18	14	14	11	6	6	0	0	0	0	0	5	5	0	4	4	7	7	7	13
C5.1	D1	microbiota+ |virus +		18	18	15	15	12	5	5	0	0	0	0	0	7	8	0	3	3	6	6	6	10
C5.1	D2	microbiota+ |virus +		18	16	13	12	10	3	1	0	2	3	3	3	12	12	0	3	3	5	5	5	6
C5.1	D3	microbiota+ |virus +		18	17	15	14	11	1	0	0	0	0	1	1	12	12	0	3	3	6	6	6	6
C5.1	D4	microbiota+ |virus +		18	18	14	14	11	6	6	0	1	1	1	2	6	6	0	3	3	6	6	6	12
CG1	G1	microbiota+ |virus +		18	18	15	15	12	2	1	0	0	0	0	0	11	12	0	3	3	6	6	6	6
CG1	G2	microbiota+ |virus +		18	18	15	15	12	1	1	0	0	0	0	0	11	12	0	3	3	6	6	6	6
CG1	G3	microbiota+ |virus +		18	18	15	14	11	1	1	0	0	0	1	1	11	12	0	3	3	6	6	6	6
CG1	G4	microbiota+ |virus +		18	18	14	14	11	2	2	0	0	0	0	0	9	10	0	4	4	7	7	7	8
C5.1	A1	microbiota- |virus-		18	17	14	12	10	6	6	0	1	1	3	3	7	7	0	3	3	5	5	5	11
C5.1	A2	microbiota- |virus-		18	16	12	12	10	3	2	0	2	2	2	2	10	10	0	4	4	6	6	6	8
C5.1	A3	microbiota- |virus-		18	18	15	14	11	8	8	0	0	0	1	1	4	4	0	3	3	6	6	6	14
C5.1	A4	microbiota- |virus-		18	17	14	14	11	11	11	0	1	1	1	1	1	1	0	3	3	6	6	6	17
CG1	E1	microbiota- |virus-		18	18	15	14	11	2	2	0	0	0	1	1	10	10	0	3	3	6	6	6	8
CG1	E2	microbiota- |virus-		18	18	15	15	12	3	3	0	0	0	0	0	9	11	0	3	3	6	6	6	7
CG1	E3	microbiota- |virus-		18	18	15	15	12	6	5	0	0	0	0	0	7	11	0	3	3	6	6	6	7
CG1	E4	microbiota- |virus-		18	18	15	14	11	7	7	0	0	0	1	1	5	8	0	3	3	6	6	6	10
C5.1	B1	microbiota- |virus+		18	15	13	13	10	1	1	0	3	3	3	4	12	12	0	2	2	5	5	5	6
C5.1	B2	microbiota- |virus+		18	18	15	13	10	0	0	0	0	1	2	2	12	12	0	3	3	6	6	6	6
C5.1	B3	microbiota- |virus+		18	17	13	13	10	3	1	0	2	2	3	4	12	12	0	3	3	5	5	5	6
C5.1	B4	microbiota- |virus+		18	17	14	12	10	2	1	0	1	2	2	2	11	12	0	3	4	6	6	6	6
CG1	F1	microbiota- |virus+		18	18	15	14	11	0	0	0	0	0	1	1	12	12	0	3	3	6	6	6	6
CG2	F2	microbiota- |virus+		18	18	15	14	1	0	0	0	0	1	12	12	13	13	0	3	3	5	5	5	5
CG1	F3	microbiota- |virus+		18	18	15	15	12	2	2	0	0	0	0	1	10	11	0	3	3	6	6	6	7
CG1	F4	microbiota- |virus+		18	18	15	15	11	1	1	0	0	0	1	1	11	12	0	3	3	6	6	6	6
			**Total**	576	562	459	444	342	139	126	0	16	20	43	47	257	280	0	101	102	191	191	193	296
