# Supplementary material for: The Gut Microbiota Can Provide Viral Tolerance in the Honey Bee
Source: Microorganisms. 2021 Apr 17;9(4):871. doi: 10.3390/microorganisms9040871 (PMC8072606; doi:10.3390/microorganisms9040871)
Supplement: Supplementary file 1 [file microorganisms-09-00871-s001.zip › microorganisms-1182355-supplementary/Supplementary material/Dosch et al. supplementary material Table S2.pdf]

Table S2. Primers used in this study and standard curve characteristics.

Target	Name	Sequence 5'-3'	Application	Amplicon size	T_m (°C)[a]	Reference	Standard curve	
							Efficiency, R^2	Slope,intercept
DWV-B	VDVq-R2a	CTT CCT CAT TAA CTG AGT TGT TGT C	qPCR: viral detection and quantification	140 bp	62	McMahon et al. 2015 [a]	93%, 0.915	-3.502, 39.994
	VDVq-F2	TAT CTT CAT TAA AAC CGC CAG GCT			63			
DWV-B	VDVq-F1a	GAA AAC ATT TGG AAT TAG CAA CGA C	PCR: external DNA standard creation	338 bp	63	McMahon et al. 2015 [a]		
	DWVDV_7AR	AAT CCG TGA ATA TAG TGT GAG G			58			
DWV-A	DWVq-F2	TGT CTT CAT TAA AGC CAC CTG GAA	qPCR: viral detection	338 bp	61	McMahon et al. 2015 a		
	DWVq-R2a	TTT CCT CAT TAA CTG TGT CGT TGA T			58			
BQCV	BQCV-qF7893	AGT GGC GGA GAT GTA TGC	qPCR: viral detection	294 bp	56	Locke et al. 2012 [b]		
	BQCV-qB8150	GGA GGT GAA GTG GCT ATA TC			58			
CBPV	CBPV1-qF1818	CAA CCT GCC TCA ACA CAG	qPCR: viral detection	296 bp	56	Locke et al. 2012 [b]		
	CBPV1-qB2077	AAT CTG GCA AGG TTG ACT GG			58			
SBV	SBV-qB3461	GCT CTA ACC TCG CAT CAA C	qPCR: viral detection	335 bp	57	Locke et al. 2012 [b]		
	SBV-qF3164	TTG GAA CTA CGC ATT CTC TG			56			
SBPV	SBPV-F3177	GCG CTT TAG TTC AAT TGC C	qPCR: viral detection	226 bp	55	de Miranda et al. 2010 [c]		
	SBPV-B3363	ATT ATA GGA CGT GAA AAT ATA C			53			
β-Actin	Am-Actin2-qF	CGT GCC GAT AGT ATT CTT G	qPCR: control viral detection and quantification/	271 bp	55	Locke et al. 2012 [b]		
	Am-Actin2-qB	CTT CGT CAC CAA CAT AGG			54			
Lactobacillus Firm-4	Lacto. Firm-4_F	AGTCGAGCGCGGGAAGTCA	qPCR: microbiota abundance quantification/external DNA standard creation	169 bp	61.6	Kesnerova et al., 2017 [d]	103.4%, 0.944	-3.243, 35.851
	Lacto. Firm-4_R	AGCCGTCTTTCAACCAGCACT			61.2			
Lactobacillus Firm-5	Lacto. Firm-5_F	GCAACCTGCCCTWTAGCTTG	qPCR: microbiota abundance quantification/external DNA standard creation	118 bp	60.5	Kesnerova et al., 2017 [d]	108.7%, 0.963	-3.129, 35.761
	Lacto. Firm-5_R	GCCCATCCTKTAGTGACAGC			60.5-62.5			
Gilliamella apicola	prPE150	CTT TGT TGC CAT CGG TTA GGC C	qPCR: microbiota abundance quantification/external DNA standard creation	160 bp	64.2	Engel P. et al. 2015 [e]	106.1%, 0.963	-3.183, 35.821
	prPE153	CCG CTT GCT CTC GCG AGG			62.9			
Frischella perrera	Frisch.perrara_F	GGA AGT TAT GTG TGG GAT AAG C	qPCR: microbiota abundance quantification/external DNA standard creation	185 bp	60.1	Kesnerova et al., 2017 [d]	100.5%, 0.976	-3.309, 36.805
	Frisch.perrara_R	CTA TTC TCA GGT TGA GCC CG			60.5			
Bartonella apis	Bart.apis_F	GTG GGA ATC TAC CTA TTT CTA CG	qPCR: microbiota abundance quantification/external DNA standard creation	103 bp	60.9	Kesnerova et al., 2017 [d]	107.3%, 0.971	-3.160, 35.417
	Bart.apis_R	AAC GCG GGC TCA TCT ATC TC			60.5			
Bifidobacterium asteroides	Bifi.aster_F	ATG CAA GTC GAA CGG GAT CC	qPCR: microbiota abundance quantification/external DNA standard creation	174 bp	60.5	Kesnerova et al., 2017 [d]	95.6%, 0.987	-3.432, 37.878
	Bifi.aster_R	CAT CCC ATR CCG GTA AAC CC			60.5-62.5			
Snodgrassella alvi	S.alvi_PE_F	CTT AGA GAT AGG AGA GTG CC	qPCR: microbiota abundance quantification/external DNA standard creation	132 bp	60.1	Kesnerova et al., 2017 [d]	98.9%, 0.965	-3.348, 35.894
	S.alvi_PE_R	AAC TTA ATG ATG GCA ACT AA			60.1			
Universal bacteria (16S rRNA)	U16SRT-F	ACT CCT ACG GGA GGC AGC AGT	qPCR: microbiota abundance quantification/external DNA standard creation	180 bp	65	Clifford et al. 2012 [f]	104.1%, 0.967	-3.227, 33.846
	U16SRT-R	TAT TAC CGC GGC TGC TGG C			62			

[a] McMahon, D.P., Furst, M.A., Caspar, J., Theodorou, P., Brown, M.J.F., and Paxton, R.J. (2015) A sting in the spit: widespread cross-infection of multiple RNA viruses across wild and managed bees. *Journal of Animal Ecology* **84**: 615-624.

[b] Locke, B., Forsgren, E., Fries, I., and de Miranda, J.R. (2012) Acaricide treatment affects viral dynamics in *Varroa destructor*-infested honey bee colonies via both host physiology and mite control. *Applied and Environmental Microbiology* **78**: 227-235.

[c] de Miranda, J.R., Dainat, B., Locke, B., Cordoni, G., Berthoud, H., Gauthier, L. et al. (2010) Genetic characterization of slow bee paralysis virus of the honeybee (*Apis mellifera* L.). *Journal of General Virology* **91**: 2524-2530.

[d] Kesnerova, L., Mars, R.A.T., Ellegaard, K.M., Troilo, M., Sauer, U., and Engel, P. (2017) Disentangling metabolic functions of bacteria in the honey bee gut. *PloS Biology* **15**: e2003467.

[e] Engel, P., Bartlett, K.D., and Moran, N.A. (2015) The bacterium *Frischella perrara* causes scab formation in the gut of its honeybee host. *mBio* **6**: e00193-00115.

[f] Clifford, R.J., Milillo, M., Prestwood, J., Quintero, R., Zurawski, D.V., Kwak, Y.I. et al. (2012) Detection of bacterial 16S rRNA and identification of four clinically important bacteria by real-time PCR. *PLoS One* **7**: e48558.
