# Supplementary material for: The Gut Microbiota Can Provide Viral Tolerance in the Honey Bee
Source: Microorganisms. 2021 Apr 17;9(4):871. doi: 10.3390/microorganisms9040871 (PMC8072606; doi:10.3390/microorganisms9040871)
Supplement: Supplementary file 1 [file microorganisms-09-00871-s001.zip › microorganisms-1182355-supplementary/Supplementary material/Dosch et al. supplementary material Table S3.pdf]

Table S3. Output of linear mixed effects models for the bacterial abundance of specific members of the bee gut microbiota (*G. apicola*, *F. perrara*, *S. alvi*, *B. apis*, *B asteroids*, *Lactobacillus Firm-4*, *Lactobacillus Firm-5*) or universally all bacteria (16S). Listed are estimates, confidence intervals (CI) and associated p-values for the predictors in the models, random effects variances (between groups: σ^2 and within-groups: τ_{00}), intraclass correlation coefficient (ICC), number of groups, number of observations, marginal and conditional R^2 as well as post-hoc tests (Westfall corrected) between predictor levels.

	**16S**			***G. apicola***			***S. alvi***			***B. asteroides***			***Firm4***			***Firm5***			***F. perrara***			***B. apis***		
Predictors	*Estimates*	*CI*	*p*	*Estimates*	*CI*	*p*	*Estimates*	*CI*	*p*	*Estimates*	*CI*	*p*	*Estimates*	*CI*	*p*	*Estimates*	*CI*	*p*	*Estimates*	*CI*	*p*	*Estimates*	*CI*	*p*
(Intercept)	19.99	18.53 – 21.46	**<0.001**	18.21	14.16 – 22.27	**<0.001**	16.97	14.23 – 19.71	**<0.001**	15.37	11.69 – 19.06	**<0.001**	14.67	11.81 – 17.54	**<0.001**	17.49	14.96 – 20.02	**<0.001**	13.42	11.14 – 15.71	**<0.001**	16.83	12.16 – 21.51	**<0.001**
colony	*Reference*			*Reference*			*Reference*			*Reference*			*Reference*			*Reference*			*Reference*			*Reference*		
microbiota+	-0.38	-1.62 – 0.85	0.542	-5.12	-7.08 – -3.15	**<0.001**	0.12	-2.11 – 2.35	0.917	-0.98	-3.88 – 1.92	0.508	-0.82	-3.52 – 1.88	0.552	0.37	-2.66 – 3.40	0.812	0.38	-1.48 – 2.25	0.686	-0.31	-3.27 – 2.64	0.836
microbiota-	-2.19	-3.43 – -0.96	**<0.001**	-8.13	-10.10 – -6.17	**<0.001**	-12.77	-15.00 – -10.54	**<0.001**	-5.79	-8.69 – -2.88	**<0.001**	-6.10	-8.80 – -3.40	**<0.001**	-4.01	-7.04 – -0.98	**0.009**	-9.48	-11.34 – -7.61	**<0.001**	-11.92	-14.88 – -8.96	**<0.001**
**Random Effects**																								
σ^2	1.72			4.88			5.58			10.67			9.22			11.65			4.41			11.06		
τ_{00}	0.53_{colony}			7.15_{colony}			2.01_{colony}			4.02_{colony}			1.64_{colony}			0.00_{colony}			1.45_{colony}			8.21_{colony}		
ICC	0.24			0.59			0.26			0.27			0.15						0.25			0.43		
N	2_{colony}			2_{colony}			2_{colony}			2_{colony}			2_{colony}			2_{colony}			2_{colony}			2_{colony}		
Observations	38			39			38			39			39			39			39			39		
Marginal R^2 / Conditional R^2	0.294 / 0.462			0.415 / 0.763			0.845 / 0.886			0.310 / 0.499			0.414 / 0.502			0.280 / NA			0.801 / 0.850			0.638 / 0.792		

Post-hoc tests (Westfall corrected)

Comparison	*Estimates*	*p*	*Estimates*	*p*	*Estimates*	*p*	*Estimates*	*p*	*Estimates*	*p*	*Estimates*	*p*	*Estimates*	*p*	*Estimates*	*p*
colony vs. microbiota+	-0.38	0.542	-5.12	**<0.001**	0.23	0.834	-0.98	0.508	-0.82	0.552	0.37	0.812	0.38	0.686	-0.31	0.836
colony vs. microbiota-	-2.19	**<0.001**	-8.13	**<0.001**	-13.34	**<0.001**	-5.79	**<0.001**	-6.10	**<0.001**	-4.01	**0.010**	-9.48	**<0.001**	-11.92	**<0.001**
microbiota+ vs. microbiota-	-1.81	**<0.001**	-3.01	**<0.001**	-13.57	**<0.001**	-4.81	**<0.001**	-5.28	**<0.001**	-4.38	**0.001**	-9.86	**<0.001**	-11.61	**<0.001**
