# Supplementary material for: The Gut Microbiota Can Provide Viral Tolerance in the Honey Bee
Source: Microorganisms. 2021 Apr 17;9(4):871. doi: 10.3390/microorganisms9040871 (PMC8072606; doi:10.3390/microorganisms9040871)
Supplement: Supplementary file 1 [file microorganisms-09-00871-s001.zip › microorganisms-1182355-supplementary/Supplementary material/Dosch et al. supplementary material Table S4.pdf]

Table S4. Output of Cox mixed effects model with bee survival in the treatment groups microbiota presence (microbiota+, microbiota-), virus presence (virus+, virus-) and their interaction as fixed factor and source colony and cage as random factor. Given are the estimates (β), the hazard ratio (Exp. β) the standard error (s.e.), the z-value and the p-value compared with the baseline treatment level (microbiota+|virus-). For random variables the standard deviation (s.d.) and the variance are given.

	coefficients				
**parameters**	β	Exp. (β)	s.e. (β)	z-value	p-value
**fixed variable**					
microbiota presence	0.69	1.98	0.30	2.26	0.024
virus presence	1.40	4.05	0.30	4.71	<0.001
interaction microbiota * virus presence	-0.13	0.88	0.4	-0.31	0.750
**random variable**	s.d.	variance			
cage	0.439	0.193			
colony	0.361	0.130			
