# Supplementary material for: The Gut Microbiota Can Provide Viral Tolerance in the Honey Bee
Source: Microorganisms. 2021 Apr 17;9(4):871. doi: 10.3390/microorganisms9040871 (PMC8072606; doi:10.3390/microorganisms9040871)
Supplement: Supplementary file 1 [file microorganisms-09-00871-s001.zip › microorganisms-1182355-supplementary/Supplementary material/Dosch et al. supplementary material Table S5.pdf]

Table S5. Output of linear mixed effects models of viral titre in either virus+ bees (groups: microbiota+|virus+ and microbiota-|virus+) or virus- bees (groups: microbiota+|virus- and microbiota-|virus-). Listed are estimates, confidence intervals (CI) and associated p-values for the predictors in the models, random effects variances (between groups: σ^2 and within-groups: τ_{00}), intraclass correlation coefficient (ICC), number of groups , number of observations, marginal and conditional R^2.

	Virus titre in virus+ bees			Virus titre in virus- bees		
Predictors	*Estimates*	*CI*	*p*	*Estimates*	*CI*	*p*
(Intercept)	51908285714.29	12952952974.68 – 90863618453.89	**0.009**	16650.00	-44046.53 – 77346.53	0.591
microbiota-virus+	*Reference*					
microbiota+virus+	9366089285.71	-43975696905.79 – 62707875477.22	0.731			
microbiota-virus-				*Reference*		
microbiota+virus-				27991.25	-1671.02 – 57653.52	0.064
**Random Effects**						
σ^2	2765258235946428530628.00			916162810.00		
τ_{00}	0.00 $_{colony}$			1689016121.45 $_{colony}$		
ICC				0.65		
N	2 $_{colony}$			2 $_{colony}$		
Observations	15			16		
Marginal R^2 / Conditional R^2	0.008 / NA			0.074 / 0.674		
